# Recommendations from long-term care reports, commissions, and inquiries in Canada

**DOI:** 10.12688/f1000research.43282.1

**Published:** 2021-02-10

**Authors:** Eric K. C. Wong, Trina Thorne, Carole Estabrooks, Sharon E. Straus

**Affiliations:** 1 Knowledge Translation Program, Li Ka Shing Knowledge Institute, St. Michael’s Hospital, Toronto, Ontario, M5B 1W8, Canada; 2 Institute for Health Policy Management and Evaluation, Dalla Lana School of Public Health, University of Toronto, Toronto, Ontario, Canada; 3 Faculty of Nursing, University of Alberta, Edmonton, Alberta, T6G 1C9, Canada; 4 Translating Research in Elder Care (TREC) program, University of Alberta, Edmonton, Alberta, T6G 1C9, Canada

**Keywords:** long-term care, nursing home, older adults, health policy, research waste, Canada, commissions, inquiries, cost analysis, quantitative analysis, COVID-19

## Abstract

**Background:** Multiple long-term care (LTC) reports have issued similar recommendations for improvement across Canadian LTC homes. Our primary objective was to identify the most common recommendations made over the past 10 years. Our secondary objective was to estimate the total cost of studying LTC issues repeatedly from 1998 to 2020.

**Methods:** The qualitative and cost analyses were conducted in Canada from July to October 2020. Using a list of reports, inquiries and commissions from The Royal Society of Canada Working Group on Long-Term Care, we coded recurrent recommendations in LTC reports. We contacted the sponsoring organizations for a cost estimate, including direct and indirect costs. All costs were adjusted to 2020 Canadian dollar values.

**Results:** Of the 80 Canadian LTC reports spanning the years of 1998 to 2020, 24 (30%) were based on a national level and 56 (70%) were focused on provinces or municipalities. Report length ranged from 4 to 1491 pages and the median number of contributors was 14 (interquartile range, IQR, 5–26) per report. The most common recommendation was to increase funding to LTC to improve staffing, direct care and capacity (67% of reports). A median of 8 (IQR 3.25–18) recommendations were made per report. The total cost for all 80 reports was estimated to be $23,626,442.78.

**Conclusions:** Problems in Canadian LTC homes and their solutions have been known for decades. Despite this, governments and non-governmental agencies continue to produce more reports at a monetary and societal cost to Canadians.

##  Introduction

The COVID-19 pandemic led to a high proportion of deaths in Canadian long-term care homes (LTCH) compared with those of other developed countries. The proportion of deaths from COVID-19 in LTCH in Canada was 81% compared with a mean of 42% in other Organisation for Economic Cooperation and Development (OECD) countries in the initial months of the pandemic ^1^. This statistic is surprising since Canada is considered to have a relatively low number of COVID-19 deaths overall ^2^. The difference in mortality rate was attributed to pandemic preparedness, integration of services (LTCH, public health and hospitals), funding and resources, daily care hours for residents, and comprehensiveness of inspections ^3^.

During the early months of COVID-19, the media reported a shortage of direct care providers and personal protective equipment (PPE) in Canadian LTCHs, which led to residents suffering from a lack of basic personal care and delays in identifying medical problems ^4^. Reduced staffing levels and wage compression in Canada’s LTC sector compelled individuals to work at more than one facility to make a living wage. Working at multiple nursing homes contributed to the spread of COVID-19 infection ^5^, and although restricting employment to one facility reduced the number of outbreaks, it also exacerbated pre-existing work force shortages ^6^. Furthermore, lower staffing levels and direct care hours are associated with increased rates of infection and hospital admission among residents ^7^, with this same trend observed in LTCHs with COVID-19 outbreaks ^3, 5, 8^.

In April, the Royal Society of Canada Working Group on Long-Term Care was tasked with reviewing the current state of LTC in the face of COVID-19 ^9^. Their report found 103 LTC reports, commissions, and inquiries, 80 of which were unique reports based in Canada. The review found recurrent themes of longstanding deficiencies in the LTC sector that contributed to the magnitude of the COVID-19 crisis in LTCHs. Despite the wealth of evidence, policy changes were not made. Instead, new LTC commissions and inquiries are being held in response to the pandemic, only to find the same problems already known from past reports ^10– 12^. Given the number of reports in the past, it would be helpful to know which recommendations were made recurrently. Furthermore, the cost of studying LTC using reports, commissions and inquires in Canada is also not known.

The primary objective of this study was to examine the recurring recommendations over the past 10 years. Our secondary objective was to calculate the total costs of generating all of the LTC reports, commissions, and inquiries in Canada from 1998 to 2020. We aimed to put the cost of repeatedly studying the same problems into context of the current pandemic.

## Methods

Quantitative analyses were based on Canadian LTC reports and commissions identified from The Royal Society of Canada Working Group on Long-Term Care ^9^, which was done using a environmental scan from 1998 to 2020, a hand search of the report citations and the identification of reports during communication with the affiliated organizations. Online resources including Google Scholar, government, professional regulatory organizations, unions, and association websites were searched using terms such as “long-term care”, “nursing home”, “residential care”, “report”, “commission”, “recommendation, and “inquiry”. The reports were sponsored by various governmental and nongovernmental organizations and authored by researchers in the field. Even though the reports are not peer-reviewed, the Royal Society working group reviewed the reports and found them to be an accurate reflection of the state of LTC ^9^.

Our analysis took place from July to October 2020. Recommendations were identified from reports published in the past 10 years to increase relevance to current practice. We only counted recommendations that were specifically stated, either in a heading, in a list, or in a “recommendations” section of the report. After the first five reports were reviewed by two reviewers, a discussion was held to decide on grouping of the recommendations. The same two reviewers regularly discussed the recommendation categories through the review process. Recommendation categories were chosen to reflect meaningful differences in concept. For example, even though improving staffing was a common theme, we separated out the recommendations into (i) increasing funding for more staff, (ii) improving staff education and training, (iii) optimizing the mix of staff professions, and (iv) increasing emotional and wellness supports for staff. For the cost analysis, we excluded follow-up reports and included cost estimates in the original larger report.

An earlier version of this article can be found on medRxiv (DOI: https://doi.org/10.1101/2020.11.17.20233114).

### Estimation of report cost

We contacted the author or sponsoring organizations of the 80 Canadian LTC reports to inquire about the estimated cost of producing each report. Using a standardized email or script for telephone calls, we requested both direct and indirect costs. Direct costs included consultancy fees, salaries, compensation for expert witnesses, graphics, layout, printing, and dissemination. Indirect costs included time donated from authors who volunteered their time to produce the report. In some cases, multiple conversations with the sponsoring organization were required to gather all of the pertinent details. When an estimated budget was not available, we searched online for global budget reports from the sponsoring organization. Total annual expenses for research, advocacy, or reports were divided by the number of reports published that year by the organization to generate an estimated cost. We also searched for media reports about costs of commissions or coroner’s inquests. If no costs were available, the estimate was based on length, depth of research, inclusion of external experts/witnesses, and the reported cost of similar LTC reports. Costs were in Canadian dollars and adjusted to 2020 values according to the Bank of Canada Inflation Calculator ^13^. 

### Data extraction and analysis

Report characteristics were extracted, including title, sponsoring organization, publication year, geographic region, scope of report, number of contributors, number of pages, and duration of the project. The sponsoring organization was defined as the group funding the report. The geographic region of the report was categorized into national, provincial, or municipal jurisdictions. We extracted the specific province or municipality. The report scope was categorized into one of the following: health system, care of older adults, continuing care (home care, assisted living, and LTC) and LTC only. The number of contributors was the total of unique authors, researchers, panel members, witnesses, and consultants, depending on the report type. The project duration was defined as the period from the date of project initiation to report completion. Data extraction and content analysis was completed by EW and TT. Accuracy of the characteristics was reviewed by both investigators and agreement on the content analysis were confirmed by discussion. Descriptive statistics incluing cost data were presented as means (standard deviation), median (interquartile range), and proportion, as appropriate. The primary outcome was recurrent recommendations. Secondary outcomes included contributors, total costs of producing the reports and median page count.

### Ethics approval

No ethics approval was required for this analysis.

## Results

**Figure 1.**
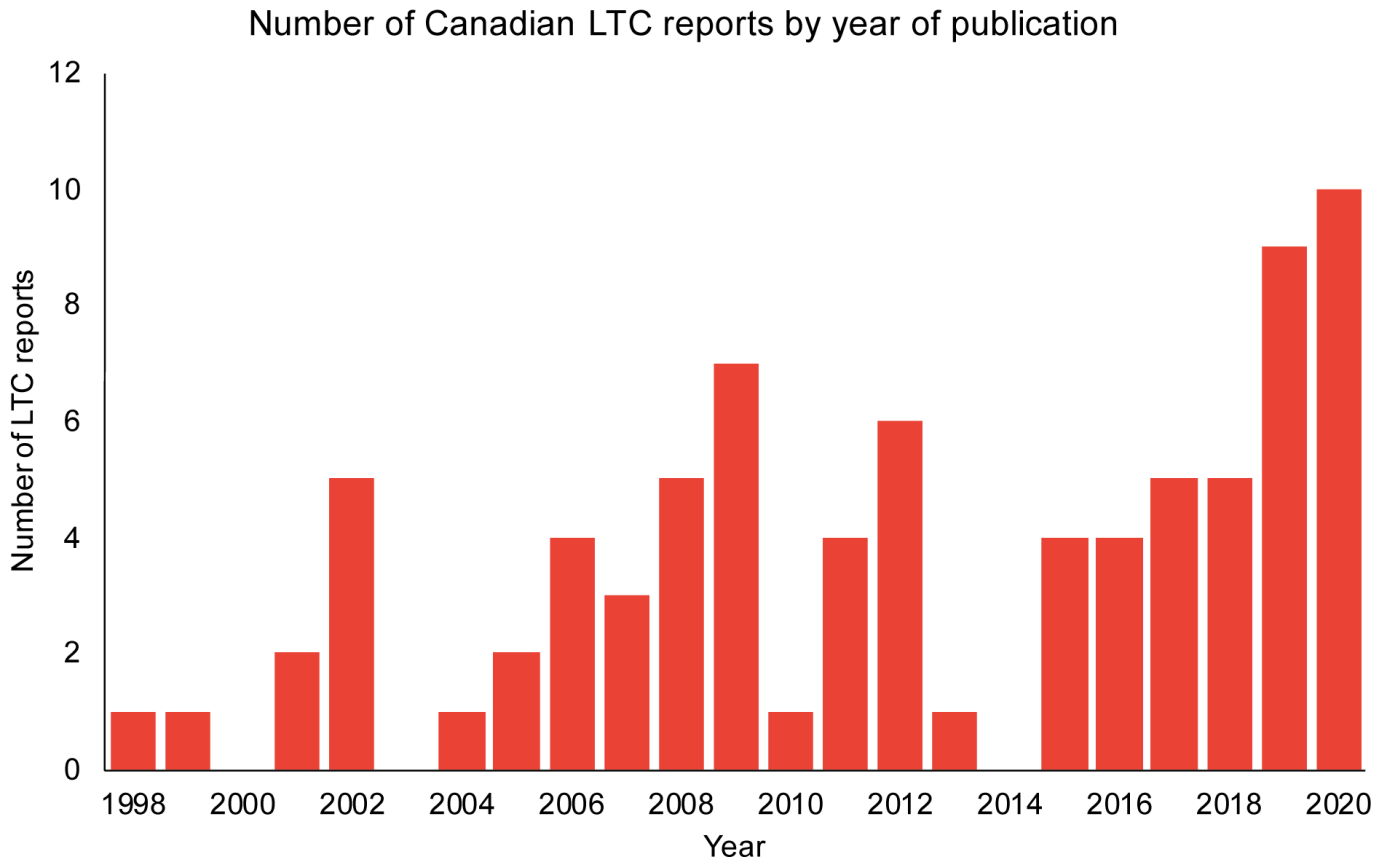
The number of Canadian reports with long-term care (LTC) recommendations over the span of 1998–2020.

The list of Canadian LTC reports from the Royal Society commission spans the years 1998 to 2020 (n=80). There was an increase in the number of reports over time, with 10 reports in the first half of 2020 ( Figure 1). Most of the reports were focused at a provincial level (n=55, 68.8%), 24 reports were based on a national level (30.0%). We found one municipal report and no reports from the territories. Ontario (n=31, 55.0%) produced the majority of the provincial reports, followed by British Columbia (n=11, 19.6%) ( Table 1). More reports were funded by provincial governments (n=26) compared with the federal government (n=9). Non-profit organizations (e.g. Canadian Association for Long Term Care, Canadian Institute of Health Information) and professional unions (e.g. Canadian Federation of Nurses Unions, Canadian Union of Public Employees) or associations (e.g. Registered Nurses' Association of Ontario, Canadian Medical Association) authored the remaining 45 reports. No reports were funded by the private sector. Most of the reports focused solely on LTC (68.8%), but some reports focused on continuing care, older adults, or the health care system as well. The median report length was 40 pages (interquartile range, IQR, 21–84), with 16 reports (20.0%) over 100 pages. The median number of contributors, including authors, witnesses, and consultants, was 14 (IQR 5–26).

**Table 1.** Report characteristics for 80 long-term care (LTC) reports in Canada from 1998–2020. The year of publication, geographic focus, funding organization, scope (topics covered), page count and number of contributors were extracted for each report. Costs were adjusted to 2020 Canadian dollar values. IQR, interquartile range.

Characteristic	Value	
Year of publication, n (%)		
2010–2020 <2010	32 (40.0) 48 (60.0)	
Geographic focus of report, n (%)		
National Ontario British Columbia Alberta Manitoba New Brunswick Nova Scotia Quebec Prince Edward Island Saskatchewan	24 (30.0) 31 (38.7) 11 (13.7) 4 (5.0) 2 (2.5) 2 (2.5) 2 (2.5) 2 (2.5) 1 (1.3) 1 (1.3)	
Funding organization *, number of reports ($ spent)		
Federal government Provincial government National non-profit Provincial non-profit Professional association Professional union Research network Private sector	9 26 13 6 20 11 1 0	$4,461,570.04 $18,270,601.07 $389,067.54 $138,651.74 $196,888.44 $169,663.95 $67,753.00 $0
Total cost (80 reports)		$23,626,442.78
Scope of report, n (%)		
Health system Older adults in general Continuing care (home care, assisted living, and LTC) LTC only	7 (8.7) 10 (12.5) 8 (10.0) 55 (68.8)	
Page count, median (IQR)	40 (20.8–84.2)	
Number of contributors for report, including witnesses and consultants, median (IQR)	14 (5–26)	

*Some reports had more than 1 funding organization.

### Common recommendations in reports

Reviewing the reports from the last 10 years (n=48), we identified a median of 8 (IQR 3.25–18) recommendations per report. Numerous recommendations were repeated in these reports ( Table 2). Overall, the most frequent recommendations were: (i) to increase or redistribute funding to improve staffing, direct care, and capacity (66.7%), (ii) to standardize, regulate and audit LTC quality of care (58.3%), and (iii) to standardize, regulate or reform education and training for LTC staff (52%). Improving staff education and training, increasing behavioural supports, and modernizing infection control measures were universally recommended in reports by governments, non-profits, professional association and unions.

**Table 2.** Recurrent recommendations in long-term care (LTC) reports in the years 2010-2020 (n=48). The full list of reports is available in *Extended data*, Supplementary Tables 1 and 2 ^14^.

Recommendation	Number of reports (%)	Reports (see supplementary table 1 for list)
**Funding**: Increase or redistribute funding to improve staffing, direct care, and capacity	32 (66.7)	1, 2, 6-12, 16, 17, 19, 21-24, 26-29, 31, 32, 34, 37, 40-44, 46-48
**LTC quality of care**: Standardize, regulate or audit LTC quality of care	28 (58.3)	1, 2, 9-12, 14, 17, 18, 20-23, 26, 27, 29, 30, 34, 35, 39, 40, 42-44, 45-48
**Education and training**: Standardize, regulate or reform education and training for LTC staff, contractors, or agency staff	25 (52.1)	2, 8, 10-12, 17, 19-21, 23, 24, 26, 28, 29, 32, 34, 37, 39, 41-44, 46-48
**Staffing mix**: Standardize, regulate or reform staffing mix in LTC	23 (47.9)	2, 7, 8, 12, 15, 16, 18, 19, 21, 23, 24, 26, 28, 29, 31, 34, 37, 39-41, 44, 46, 48
**Data driven**: Collect, standardized, or use data or evidence to improve LTC	22 (45.8)	1, 7, 9, 11, 12, 18, 19, 21, 23, 24, 27, 29, 30, 35, 37, 38-40, 42, 43, 46, 47
**Staff support**: Increase resources or funding for LTC staff emotional support, enhancement of well being, supporting tuition/ training, supporting staff family/dependents.	16 (33.3)	2, 8, 11, 12, 16, 19, 20, 21, 23, 24, 26, 34, 37, 43, 44, 47
**Behaviours**: Increase support, staffing, resources, or training for patients with responsive behaviours	15 (31.3)	7, 8, 11, 12, 17, 26, 28, 32, 37, 42, 43, 46-48
**Federal involvement**: Standardize, regulate, or increase funding from the federal level	11 (22.9)	2, 6, 11, 12, 21, 23, 27, 30, 34, 40, 47
**For-profit homes**: Regulate, reduce, or reform for-profit LTC homes	11 (22.9)	1, 2, 9, 20-22, 34, 40, 46-48
**Critical incidents**: Report, track, or improve transparency about critical incidents in LTC	9 (18.8)	5, 11, 14, 21, 28, 32, 35, 43, 46
**Commission**: Appointment of a commission, task force, committee or inquiry	9 (18.8)	6, 11, 15, 31, 34, 40, 41, 43, 46,
**Infection control**: National standard for LTC infection control protocol, PPE, or staffing/visitor policies during outbreaks	5 (10.4)	2, 6, 10, 14, 46

Government-authored reports (n=35) focused on improving data collection (n=8, 22.9%), improving education and training of staff (n=8, 22.9%) and standardizing LTC quality of care (n=8, 22.9%). Professional union reports (n=11) focused on regulating for-profit LTCHs (n=4, 36.4%) and standardizing LTC quality of care (n=4, 36.4%). The most common recommendation made by non-profits (n=19) was to improve training and education for staff (n=9, 47.4%). Professional association reports (n=20) most commonly recommended standardizing staffing mix (n=10, 50.0%) and increasing funding for direct care (n=10, 50.0%). The least common recommendation made by professional associations was for improved transparency, reporting and tracking of critical incidents (n=1, 5.0%).

Critical incidents received the most attention from governments (n=6, 17.1%), followed by non-profit organizations (n=2, 10.5%) and a professional association (n=1, 5.0%). Recommendations regarding quality of care or data utilization were more likely to be made by government (n=18, 51.4%) or professional associations and unions (n=20, 64.5%) than by non-profits (n=3, 15.8%). Staff wellness support was most recommended by non-profits (n=7, 36.8%), followed by professional associations or unions (n=6, 19.4%), then government (n=3, 8.6%). The appointment of commissions or inquiries was mostly recommended by professional associations or unions (n=7, 22.6%) then by government (n=2, 5.7%) and no commissions were recommended by non-profit organizations

Although recommendations to improve resident quality of life were mentioned in 12 reports, they mostly overlapped with other recommendations such as increasing direct care, optimizing staff mix, increasing quality of life data collection, and improving quality of care. Only one report discussed specific recommendations to improve quality of life, such as increasing decision-making capacity (e.g. choice over when to bath), improving privacy with single rooms, and prioritizing relational care over medical tasks and interventions ^15^.

The full list of reports is available in *Extended data*, Supplementary Table 1 ^14^

### Cost of reports

Nearly half of the reports (45%) had cost estimates by the sponsoring organization or by publicly available budget or media reports. The total cost for all 80 reports was estimated to be $23,626,442.78 in Canadian dollars inflated to 2020 values ( Table 1). The median cost per report was $15,203.48 (IQR $10,000–$53,147.81). Details of each report’s cost estimate are shown in *Extended data*, Supplementary Table 2 ^14^. The lowest cost was estimated at $500 for each of the Canadian Army Joint Task Force reports, which accounts for the administrative costs of writing the reports even if the services were provided on military order ^4^. If the cost of military deployment was included, the total cost would increase by $53 million ^16^. The highest cost was $9,046,255.51 for the Public Inquiry into the Safety and Security of Residents in the Long-Term Care Homes System (The Wettlaufer Report) in 2019, which involved 79 contributors, witnesses, and experts ^17^.

## Discussion

This analysis highlights the efforts of multiple organizations, both governmental and non-governmental, to address the longstanding challenges and quality issues in Canada’s LTCHs. The Royal Society Briefing Report concluded that the unresolved issues in Canada’s nursing homes have been known for decades. According to our analysis, there are numerous recommendations backed by governmental and nongovernmental groups, yet little action has followed. This inaction set the stage for increased deaths during COVID-19 pandemic and to lower quality of life in LTCHs. Our analysis further showed the substantial cost of studying the problem repeatedly over the years.

Duplicate investigation of known findings reduces value and increases waste ^18^. From the LTC reports, issues regarding understaffing, undertraining, and the negative impact of for-profit LTC homes were repeatedly mentioned ^9^. Policy change often requires persistence ^19^, but the cost of advocating for change should be viewed from a societal context (financial and moral). Commissions and inquiries into LTC issues, like those happening or slated to begin around the country ^10– 12^, can solidify our determination for policy change, but do not replace the need for policy implementation. Only action will help LTC residents.

Several provinces have increased wages and provided full-time employment with more appropriate compensation and benefits to stabilize the LTC workforce ^20^. The Ontario government went further to commit 4.00 hours per day of direct care for each LTC resident by 2024 ^21^, bringing the total care hours above the national average (3.30 hours) ^22^. However, increasing direct care hours, while essential, is only one of the many recommendations from existing reports. Identifying the right staff mix and care team composition, providing proper education and training, and supporting staff wellness are also critical to developing a long-term workforce that has sufficient resilience to confront future crises ^23^. Furthermore, we should focus more attention on resident quality of life, which should be the ultimate goal of our efforts.

Although the total cost of $23 million in generating LTC reports may seem insignificant compared to a government budget, it represents a substantial, lost opportunity to continually improve Canadian LTCHs. Studying the same problems repeatedly means Canadian experts are confined to revisiting critical deficiencies in LTC instead of innovating new care models. While Canada ranks among the best countries in the world for health innovation ^24^, this pandemic highlighted a contrasting story of neglected policy in LTC ^25^.

There are several strengths of this study. We systematically tracked down the cost of each report by contacting the sponsoring organization or consulting their global budgets. The total costs, including time donated of experts authoring these reports, were accounted for. For reports that did not have available cost data, we estimated the total cost by using reports with known costs with similar length and depth.

The main limitation of this study was the lack of true cost estimates for half of the LTC reports. Some organizations lacked transparency about costing, and others lacked detailed accounting of spending. Staff turnover and record keeping practices were barriers to accessing data, particularly for the reports produced over 10 years ago. For government agencies, we often had to call multiple departments and speak with numerous representatives and noted considerable variation in the level of disclosure. For the two military reports during COVID-19, we likely underestimated the cost since they were generated as part of military duty. However, there were still considerable costs incurred by the public by having the military deployed to those LTCHs ^26^. We also grouped reports on health system improvement and care of older adults in general in this analysis because LTC is intricately tied to the health system at large. Leaders in the care of older adults, such as Denmark, design LTC policy as in integral part of their health system ^27^. 

## Conclusion

Over the last two decades, Canadian governments and non-government organizations have repeatedly investigated longstanding LTC issues and have largely drawn the same conclusions. Had the recurring recommendations been implemented, we would not only have improved working conditions, quality of care and quality of life, but would also have undoubtedly prevented unnecessary deaths due to COVID-19. Instead of continuing to investigate LTC issues, we should focus our resources on implementing the recommendations in the identified reports.

## Data availability

### Underlying data

All data underlying the results are available as part of the article and no additional source data are required.

### Extended data

Figshare: Supplementary tables from Recommendations from long-term care reports, commissions, and inquiries in Canada. https://doi.org/10.6084/m9.figshare.13537412 ^14^.

This project contains the following extended data:

- Supplementary Table 1
- Supplementary Table 2

Extended data are available under the terms of the Creative Commons Attribution 4.0 International license (CC-BY 4.0).
