# Supplementary material for: Recommendations from long-term care reports, commissions, and inquiries in Canada
Source: F1000Res. 2021 Sep 29;10:87. Originally published 2021 Feb 10. [Version 3] doi: 10.12688/f1000research.43282.3 (PMC8474099; doi:10.12688/f1000research.43282.3)
Supplement: Peer review history

# Peer review: Reviewer response for version 2

**DOI:** 10.5256/f1000research.57407.r93857

**Published:** 2021-09-24

**Authors:** Catherine E. Tong, Karen Khalil El Hajj

**Affiliations:** 1 School of Public Health Sciences, University of Waterloo, Waterloo, ON, Canada

Thank you for the opportunity to review this novel manuscript. This paper provides an overview of the numerous reports and inquiries that have been commissioned across Canada regarding long-term care (LTC) and synthesizes the most common recommendations. The authors highlight the excessive costs incurred to produce these reports and make a compelling case for turning our efforts and attention to real change, rather than continuing to report (in a costly fashion) on known issues. We offer but a few queries that the authors may wish to consider:   

- The authors spoke with organizations using a telephone script to ascertain costs. Can you please comment on why this did/did not require ethics approval?
- Regarding the sentence, “Indirect costs included time donated from authors who volunteered their time to produce the report.”: The definition of indirect costs usually entails overhead or administrative costs, yet an author’s direct time dedicated to writing up the report could be considered a direct cost, even if the author had a non-compensated role.
- As the authors note in the limitations, half of the reports’ costs had to be estimated, largely because organizations could not be contacted or would not provide a cost estimation. A significant proportion of the data was estimated by the authors. While we recognize that the calculation used (# of reports per year/cost of all reports per year) might be the only way to estimate these costs, it is entirely possible that this is not an accurate representation. For example, an organization may have easily spent 80% of their annual budget on one large report and 20% on a series of smaller reports. Perhaps an additional sentence in the “Estimation of Cost Section” is warranted?
- Table 2 mentions responsive behaviours. Would it be possible to elaborate on the definition of responsive behaviour for readers without a health research/clinical background?
- In the cost and discussion section, there is mention of military reports as well as relevant deployments to long-term care facilities. Given this journal’s international audience, it may be important to describe why there would be military reports and deployments to this sector.
- In the discussion section, the authors emphasize the costs and lost opportunities to Canada; however, LTC in Canada is managed provincially, and 31 of these reports focused on Ontario. How confident are you that the statements made in the discussion represent “Canada”, and not specific provinces? We agree that there is no need to produce further reports, but do the data presented and arguments made represent “Canada”, or do they represent the state of LTC in some provinces and territories?

Is the work clearly and accurately presented and does it cite the current literature?

Yes

If applicable, is the statistical analysis and its interpretation appropriate?

Not applicable

Are all the source data underlying the results available to ensure full reproducibility?

Yes

Is the study design appropriate and is the work technically sound?

Yes

Are the conclusions drawn adequately supported by the results?

Yes

Are sufficient details of methods and analysis provided to allow replication by others?

Yes

Reviewer Expertise:

Dr. Tong: Geriatric health systems; home and community care; review article methods. Ms. Hajj is a doctoral student and trained health economist, with specialization in LTC.

We confirm that we have read this submission and believe that we have an appropriate level of expertise to confirm that it is of an acceptable scientific standard.

# Response

**Published:** 2021-09-25

**Authors:** Eric KC Wong

**Affiliations:** University of Toronto, Canada

**Thank you for the comments. Please see below for responses to each comment.**

***The authors spoke with organizations using a telephone script to ascertain costs. Can you please comment on why this did/did not require ethics approval?***

**Author response: **Ethics approval was not needed because we did not have research participants. The phone calls and emails were to gather information regarding report costs and not information regarding the staff answering the phone calls. As such, they were not research participants and ethics approval was not needed.

 This flow diagram is helpful: https://uwaterloo.ca/research/sites/ca.research/files/uploads/files/research_or_quality_assurance_decision_tree.pdf

***Regarding the sentence, “Indirect costs included time donated from authors who volunteered their time to produce the report.”: The definition of indirect costs usually entails overhead or administrative costs, yet an author’s direct time dedicated to writing up the report could be considered a direct cost, even if the author had a non-compensated role.***

**Author response: **Thank you for the correction. We have changed the line to:

 “Direct costs included time donated from authors who volunteered their time to produce the report, consultancy fees, salaries, compensation for expert witnesses, graphics, layout, printing, and dissemination. Indirect costs included administrative costs and overhead.” – Methods > Estimation of report cost, first paragraph.

***As the authors note in the limitations, half of the reports’ costs had to be estimated, largely because organizations could not be contacted or would not provide a cost estimation. A significant proportion of the data was estimated by the authors. While we recognize that the calculation used (# of reports per year/cost of all reports per year) might be the only way to estimate these costs, it is entirely possible that this is not an accurate representation. For example, an organization may have easily spent 80% of their annual budget on one large report and 20% on a series of smaller reports. Perhaps an additional sentence in the “Estimation of Cost Section” is warranted?***

**Author response: **We added a sentence to the paragraph (added sentence in bold and underlined):

 “When an estimated budget was not available, we searched online for global budget reports from the sponsoring organization. Total annual expenses for research, advocacy, or reports were divided by the number of reports published that year by the organization to generate an estimated cost. **This calculation assumed that all reports by the organization in that year were equal in cost.**” – Methods > Estimation of report cost, first paragraph.

***Table 2 mentions responsive behaviours. Would it be possible to elaborate on the definition of responsive behaviour for readers without a health research/clinical background?***

**Author response: **Added footnote to Table 2.

 “*Responsive behaviours refer to actions, words or gestures that patients with dementia express in response to internal or external stimuli. Some examples of responsive behaviours include agitation, aggression, wandering, vocalization, etc.”

 Reference: https://www.behaviouralsupportsontario.ca/32/behaviour_change_in_older_adults/

***In the cost and discussion section, there is mention of military reports as well as relevant deployments to long-term care facilities. Given this journal’s international audience, it may be important to describe why there would be military reports and deployments to this sector.***

**Author response: **Added sentence to the discussion (in bold and underlined):

 “The main limitation of this study was the lack of true cost estimates for half of the LTC reports. Some organizations lacked transparency about costing, and others lacked detailed accounting of spending. Staff turnover and record keeping practices were barriers to accessing data, particularly for the reports produced over 10 years ago. For government agencies, we often had to call multiple departments and speak with numerous representatives and noted considerable variation in the level of disclosure. For the two military reports during COVID-19, we likely underestimated the cost since they were generated as part of military duty. **The Canadian government deployed military personnel to some nursing homes due to critical shortages of staffing and resources when the pandemic started (16).** However, there were still considerable costs incurred by the public by having the military deployed to those LTCHs (26). We also grouped reports on health system improvement and care of older adults in general in this analysis because LTC is intricately tied to the health system at large. Leaders in the care of older adults, such as Denmark, design LTC policy as an integral part of their health system (27).” – Discussion, last paragraph.

***In the discussion section, the authors emphasize the costs and lost opportunities to Canada; however, LTC in Canada is managed provincially, and 31 of these reports focused on Ontario. How confident are you that the statements made in the discussion represent “Canada”, and not specific provinces? We agree that there is no need to produce further reports, but do the data presented and arguments made represent “Canada”, or do they represent the state of LTC in some provinces and territories?***

**Author response: **Although many reports were from Ontario (n=31), the majority of reports were based nationally or in other provinces (n=49). Recommendations from these reports were similar. The Royal Society of Canada “Restoring Trust” report on LTC (reference 9 in article), which was written by experts across the country, concluded that challenges to LTC were a national problem that required a concerted effort to fix. Although there may be provincial differences in LTC management, the recurrent problems are similar. A similar analysis from the Canadian Institutes of Health Information found that recommendations from COVID-19 LTC inquires from across the country came to similar conclusions (reference below). We think that the problems identified in these reports are applicable nationwide.

 Reference: https://www.cihi.ca/sites/default/files/document/impact-covid-19-long-term-care-canada-first-6-months-report-en.pdf

# Peer review: Reviewer response for version 1

**DOI:** 10.5256/f1000research.46348.r79798

**Published:** 2021-05-25

**Authors:** Adelina Comas-Herrera

**Affiliations:** 1 Care Policy and Evaluation Centre, London School of Economics, London, UK

This article is very timely, in the context of the need to learn lessons from the COVID pandemic’s impact on long-term care systems. The pandemic has drawn wider attention to the long-standing structural problems of long-term are systems, and particularly long-term care facilities.

 The analysis of the recommendations is particularly interesting, confirming that many of the problems identified during the pandemic were well known before and action was not taken to address them. I would have liked, ideally to see this separated by whether the reports were from before or during the COVID-19 pandemic, it would be interesting to highlight if, for example, infection control was present in the reports prior to the pandemic. In my view, some of the detailed reports on COVID outbreaks in specific facilities are very different than the reports on the structure of the LTC system and would need to be considered separately.

 The analysis of the costs of the reports is an important contribution, but I have not been able to check the figures properly: I have checked the supplementary table 2 using the link provided and I cannot see cost estimates for any of the reports published since 2002. It may be that the table is incomplete?

 I have a major concern on the interpretation of the costs of the reports: while I do agree that it is very important to ensure that there is no replication of reports and to avoid waste, I do not think the paper has established clearly the extent to which “the same problems have been studied repeatedly”. While coming to the same broad recommendations, some of the reports may have advanced the understanding of the system and generated new data that may had broader impacts, for example informing planning at regional level of information practice by providers.

Is the work clearly and accurately presented and does it cite the current literature?

Yes

If applicable, is the statistical analysis and its interpretation appropriate?

Partly

Are all the source data underlying the results available to ensure full reproducibility?

Partly

Is the study design appropriate and is the work technically sound?

Yes

Are the conclusions drawn adequately supported by the results?

Partly

Are sufficient details of methods and analysis provided to allow replication by others?

No

Reviewer Expertise:

Long-term care and dementia care policy and economic aspects of care.

I confirm that I have read this submission and believe that I have an appropriate level of expertise to confirm that it is of an acceptable scientific standard, however I have significant reservations, as outlined above.

# Response

**Published:** 2021-05-31

**Authors:** Eric KC Wong

**Affiliations:** University of Toronto, Canada

Thank you for reviewing the article and for providing comments. Please see our response below:

*Comment: The analysis of the recommendations is particularly interesting, confirming that many of the problems identified during the pandemic were well known before and action was not taken to address them. I would have liked, ideally to see this separated by whether the reports were from before or during the COVID-19 pandemic, it would be interesting to highlight if, for example, infection control was present in the reports prior to the pandemic. In my view, some of the detailed reports on COVID outbreaks in specific facilities are very different than the reports on the structure of the LTC system and would need to be considered separately.*

**Response We added a line highlighting that similar recommendations were found in reports dated before and after the pandemic. In Table 2, we added a line to the caption indicating that reports 1-10 were from after the pandemic and 11-48 were from before the pandemic. Each recommendation category had reports from both before and after the pandemic.**

**“Reviewing the reports from the last 10 years (n=48), we identified a median of 8 (IQR 3.25–18) recommendations per report. Numerous recommendations were repeated in these reports, and similar recommendations were made before and after the COVID-19 pandemic (Table 2).” – Results section, second paragraph**

*Comment: The analysis of the costs of the reports is an important contribution, but I have not been able to check the figures properly: I have checked the supplementary table 2 using the link provided and I cannot see cost estimates for any of the reports published since 2002. It may be that the table is incomplete?*

**Response: The figshare website only preview the first 2 pages of the document for some reason. If you download the Word document from their website, the full table is shown. Please let us know if you have problems with the file.**

*Comment: I have a major concern on the interpretation of the costs of the reports: while I do agree that it is very important to ensure that there is no replication of reports and to avoid waste, I do not think the paper has established clearly the extent to which “the same problems have been studied repeatedly”. While coming to the same broad recommendations, some of the reports may have advanced the understanding of the system and generated new data that may had broader impacts, for example informing planning at regional level of information practice by providers.*

**Response: We agree that reports may advance our understanding of the LTC system in small increments, but based on the number of times similar recommendations have been repeated, we think there is substantial overlap in problems identified in the reports. As an example, the recently published Ontario Long Term Care COVID-19 Commission had 85 recommendations** **[1]** **. Only the first 26 recommendations were about pandemic management. The remaining 59 recommendations addressed longstanding issues with the LTC system, including resident quality of life, staff shortages, lack of staff training, poor working conditions, lack of funding, inadequate regulation, insufficient accountability, and lack of performance indicators and standards. Even the first 26 recommendations included general infection control measures previously recommended during the SARS pandemic in 2003. Similarly, the authors of the included reports in our analysis chose to highlight the same broad recommendations because those are the limiting factors to improving the LTC system. Even if there were incremental lessons and improvements, the fact that recommendations were not substantively different represented a missed opportunity to correct the most important, longstanding problems.**

**Added line in discussion:**

**“Although the total cost of $23 million in generating LTC reports may seem insignificant compared to a government budget, it represents a substantial, lost opportunity to continually improve Canadian LTCHs. Studying the same problems repeatedly means Canadian experts are confined to revisiting critical deficiencies in LTC instead of innovating new care models. Even if there were incremental lessons and improvements from these reports, they were inadequate in preventing one crisis after another. While Canada ranks among the best countries in the world for health innovation, this pandemic highlighted a contrasting story of neglected policy in LTC.” – Discussion section, fourth paragraph**

 Reference

 1.        Marrocco FN, Coke A, Kitts J. Ontario’s Long-Term Care COVID-19 Commission: Final Report [Internet]. Toronto; 2021 Apr [cited 2021 May 29]. Available from: http://www.ltccommission-commissionsld.ca/report/pdf/Ontarios_Long-Term_Care_COVID-19_Commission_Final_Report.pdf

# Peer review: Reviewer response for version 1

**DOI:** 10.5256/f1000research.46348.r79291

**Published:** 2021-03-09

**Authors:** Lynn Chenoweth

**Affiliations:** 1 University of New South Wales, Sydney, Australia

This review on the breadth, costs and recommendations arising from investigative reports on the Canadian LTC system is comprehensive, insightful and plausible. 

 It reflects the situation in many other developed nations and provides sufficient evidence to taking global action to redress the continuing government failure to redress LTC staffing issues.

 This well-conducted review has clearly identified the issues associated with funding support for the aged care sector, as identified in multiple commissions of inquiry and independent reviews over a decade. The review highlights the poor-level of government sponsorship of the sector, which translates to having inadequate funds allocated to professional staffing complements. This issue has led to cuts in professional staff and an over-supply of poorly paid and inadequately-educated care workers. Specialised care and therapy needs remain unmet for the majority of older people who would benefit from receiving them. The review makes clear that the funds spent on investigating and identifying these issues have been high, and have not resulted in any tangible benefits for the sector. The authors argue that if these funds had been used to actually redress the staffing issue, the benefits would be obvious. This review has international importance, since these issues are repeated across the aged care sector in both developed and under-developed nations. Clearly, it does not need any further commissioned reviews and independent inquiries to reinforce what we already know - that care standards will only improve when aged care is funded commensurate with acute care sector services for older people.

Is the work clearly and accurately presented and does it cite the current literature?

Yes

If applicable, is the statistical analysis and its interpretation appropriate?

Yes

Are all the source data underlying the results available to ensure full reproducibility?

Yes

Is the study design appropriate and is the work technically sound?

Yes

Are the conclusions drawn adequately supported by the results?

Yes

Are sufficient details of methods and analysis provided to allow replication by others?

Yes

Reviewer Expertise:

Aged care, transitions in ageing, dementia care, heath and aged care services, person-centered care, carer support.

I confirm that I have read this submission and believe that I have an appropriate level of expertise to confirm that it is of an acceptable scientific standard.
